# Evaluation of OCT2‐mediated drug–drug interactions between ulotaront and metformin in subjects with schizophrenia

**DOI:** 10.1002/prp2.1191

**Published:** 2024-03-25

**Authors:** Guangqing Xiao, Hironobu Tsukada, Yu‐Luan Chen, Lei Shi, Seth C. Hopkins, Gerald R. Galluppi

**Affiliations:** ^1^ Sumitomo Pharma America, Inc Cambridge Massachusetts USA

**Keywords:** drug interactions, drug transporters, schizophrenia, TAAR1, ulotaront

## Abstract

Ulotaront (SEP‐363856) is a TAAR1 agonist, with 5‐HT1A agonist activity, currently in clinical development for the treatment of schizophrenia. In vitro studies indicate ulotaront is an OCT2‐specific inhibitor with IC_50_ of 1.27 μM. The primary objective of this study is to determine if a single dose of ulotaront affects the PK of metformin, an index substrate of OCT2, in subjects with schizophrenia. In a randomized, single‐blind, 2‐period crossover study, 25 adults with schizophrenia received a single dose of metformin‐HCl 850 mg (approximately 663 mg metformin) with and without coadministration of 100 mg ulotaront. The plasma samples were analyzed by fully validated LC–MS/MS methods. The primary PK endpoints for metformin were AUC_inf_, AUC_last_, *C*_max_, and *t*_max_. The highest‐anticipated clinical dose of ulotaront (100 mg) had no statistically significant effect on the PK of a single dose of metformin based on *C*_max_ and AUC_inf_. Geometric least squares mean ratios were 89.98% and 110.63%, respectively, with the 90% confidential interval (CI) for each parameter contained within 80%–125%. Median *t*_max_ was comparable across the treatments. Ulotaront does not act as a perpetrator of OCT2‐mediated DDI against metformin. Co‐administration of ulotaront is not expected to require dose adjustment of metformin or other drugs cleared by OCT2.

## Graphical abstract

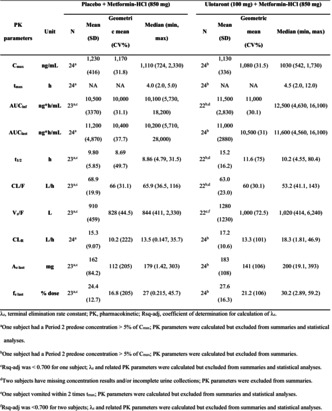

**Abbreviations**
5‐HT1A: serotonin 1A receptor
AE: adverse events
AUC: area under the curve
BCRP: breast cancer resistance protein
BSEP: bile salt export pump
*C*_max_
: maximum plasma concentration
C‐SSRS: Columbia suicide severity rating scale
CYP2D6: cytochrome P450 2D6
DDI: drug‐drug interaction
ECG: electrocardiograms
FDA: Food and Drug Administration
IM: intermediate metabolizers
LC‐MS/MS: tandem mass spectrometry
MATE: multi‐drug and toxin extrusion
NADPH: nicotinamide adenine dinucleotide phosphate
NM: normal metabolizers
OAT: organic anion transporter
OATP: organic anion transporting polypeptide
OCT: organic cation transporter
PBPK: physiologically based pharmacokinetic
P‐gp: P‐glycoprotein
PK: pharmacokinetic
SAE: severe adverse events
TAAR1: trace amine‐associated receptor 1
*t*_max_
: time of the maximum plasma concentration

## 1 INTRODUCTION

Ulotaront is a trace amine‐associated receptor 1 (TAAR1) agonist with serotonin 1A receptor (5‐HT_1A_) agonist activity. It has received US Food and Drug Administration (FDA) breakthrough therapy designation for the treatment of patients with schizophrenia and is currently in phase III clinical trials. Ulotaront has shown broad efficacy in animal models of schizophrenia (relating to positive and negative symptoms) and depression.^1^, ^2^, ^3^, ^4^ Population pharmacokinetic (PK) analysis showed that ulotaront is rapidly absorbed (median *t*_max_ of 2.8 h) and quickly cleared from plasma (median effective *t*_1/2_ of 7 h) after oral administration, and exposures increase dose‐proportionally at therapeutic dose levels ranging from 25 mg to 100 mg q.d.^5^ There is minimal accumulation, approximately 1.1‐fold after repeat dosing to steady‐state.^5^

Ulotaront elimination is mainly determined by hepatic metabolism via both nicotinamide adenine dinucleotide phosphate (NADPH)‐dependent and NADPH‐independent pathways, with Cytochrome P450 2D6 (CYP2D6) as the major metabolizing enzyme.^5^, ^6^ Ulotaront renal clearance is close to the unbound glomerular filtration rate and plays a minor role in the elimination.^5^ Drug–drug interaction (DDI) with ulotaront as the victim of CYP2D6 inhibition has been investigated in the clinic. The study indicated ulotaront area under the curve (AUC) and *C*_max_ increased by 1.72‐ and 1.32‐fold, respectively, when co‐administrated with the strong CYP2D6 inhibitor paroxetine.^7^

In vitro studies using transfected HEK293 cell lines or membrane vesicles were conducted to determine if ulotaront is a substrate or inhibitor of major human efflux and uptake transporters. The transporters that were evaluated included efflux transporters P‐glycoprotein (P‐gp), breast cancer resistance protein (BCRP), and bile salt export pump (BSEP); and uptake transporters multi‐drug and toxin extrusion 1 (MATE1), MATE2‐K, organic anion transporter 1 (OAT1), OAT3, organic cation transporter 1 (OCT1), OCT2, organic anion transporting polypeptide 1B1 (OATP1B1), and OATP1B3. The study indicated ulotaront is not a substrate of human P‐gp or BCRP, MATE1, MATE2‐K, OAT1, OAT3, OATP1B1, OATP1B3, OCT1, or OCT2. Ulotaront is not an inhibitor of the tested transporters either, except that it showed specific inhibition of OCT2 (IC_50_ = 1.27 μM) over MATEs (IC_50_ > 100 μM).^6^

SEP‐383103, a carboxylic acid metabolite of ulotaront, was identified as one of the major metabolites in preclinical species and the only major metabolite in humans.^6^ SEP‐383103 is not an inhibitor of human P‐gp, BCRP, MATE1, MATE2‐K, OAT1, OAT3, OATP1B1, OATP1B3, OCT1, or OCT2. Although SEP‐383103 showed weak inhibition on OAT3 (~35% inhibition at 50 μM) and MATE1 (~20% inhibition at 50 μM) when tested in vitro, the IC_50_ is at least more than 50‐fold greater than the unbound *C*_max_.^6^ Furthermore, SEP‐383103 is a pharmacology‐inactive metabolite. A screen of 80 known receptors, ion channels, and enzyme targets at SEP‐383103 concentrations up to 10 μM did not identify binding or functional activity (internal data).

After daily repeat dosing of 100 mg ulotaront (highest anticipated clinical dose level), the unbound *C*_max_ of ulotaront and SEP‐383103 at steady state are approximately 1.2–1.5 μM, and 1 μM, respectively (results to be published). Based on the data from clinical and in vitro studies, drug transporter‐mediated DDI with ulotaront or SEP‐383103 as the perpetrators are remote according to FDA DDI guidance, except OCT2‐mediated DDI risk is likely with ulotaront as the perpetrator.^8^ Therefore, the impact of ulotaront on OCT2 activity was investigated in a randomized, single‐blind, 2‐period crossover clinic study. A single dose of ulotaront was selected because little or no accumulation of ulotaront upon multiple dose administration was observed in human subjects.^5^ The primary objective of the study was to determine the effect of a single dose of ulotaront on the PK of a single oral dose of metformin, an index substrate of OCT2,^9^, ^10^ in subjects with schizophrenia. Adults with schizophrenia received a single dose of metformin‐HCl 850 mg (approximately 663 mg metformin) with and without co‐administration of a single dose of 100 mg ulotaront. The PK plasma samples were analyzed by fully validated liquid chromatography with tandem mass spectrometry (LC–MS/MS) methods for corresponding analytes. The primary PK endpoints for metformin are area under the plasma concentration‐time curve from time zero to infinity (AUC_inf_), area under the plasma concentration‐time curve from time zero to the last measurable concentration (AUC_last_), maximum observed plasma concentration (*C*_max_), and time of the maximum observed plasma concentration (*t*_max_).

## 2 METHODS

### 2.1 Subjects

Twenty‐five subjects were dosed in order to have at least 20 subjects complete the study. This sample size would allow at least 90% power to reject the null hypothesis that the ratio of test mean (metformin + ulotaront) to the reference mean (metformin alone) is below 0.80 or above 1.25 for the primary PK endpoints *C*_max_ and AUC_inf_. Every attempt was made to enroll at least 30% for both sexes.

Following screening evaluations, treatment with oral psychotropic medications and any other medications with a propensity for psychotropic effects were discontinued for at least 3 days or 5 half‐lives (whichever was longer) before the study.

Since ulotaront metabolism is determined by CYP2D6, CYP2D6 genotyping for all subjects were performed and only normal metabolizers (NM) or intermediate metabolizers (IM) were enrolled in the study.

### 2.2 Trial objectives, design and treatments

The study was a randomized, single‐blind, 2‐period crossover study. All subjects were randomized equally to 1 of 2 treatment sequences. Treatment Sequence 1 was single doses of placebo and 850 mg metformin‐HCl followed 5 days later by single doses of 100 mg ulotaront and metformin. Treatment Sequence 2 was single doses of 100 mg ulotaront and 850 mg metformin‐HCl followed 5 days later by single doses of placebo and 850 mg metformin‐HCl. In both Sequences 1 and 2, placebo or ulotaront was dosed 1 h prior to metformin administration and 30 min prior to the meal administration.

A stratified randomization was utilized to ensure treatment sequence balance within sex and site. Blood and urine PK samples were obtained during each treatment period. Subjects resumed treatment with their antipsychotic medication as clinically indicated for at least 3 days (restabilization period) prior to discharge from the clinical research site. Subjects were required to return 7 days (±2 days) after clinic discharge for a follow‐up visit.

### 2.3 Pharmacokinetics

Serial blood samples for determination of plasma metformin, ulotaront, and SEP‐383103 (the major metabolite of ulotaront) concentrations were collected from Day 1 to Day 4 and Day 6 to Day 9 at pre‐dose (right before study drug administration; ulotaront or placebo) as well at 0, 0.5, 1, 2, 3, 4, 5, 7, 12, 24, 36, 48, 54, and 72 h post‐dose of metformin. Urine samples were collected at pre‐dose (within 30 min prior to administration of ulotaront or placebo), and 0–24, 24–48, and 48–72 h post‐metformin administration.

The primary PK endpoints for all analytes were AUC_inf_ and *C*_max_. The PK analysis was conducted using Phoenix WinNonlin (version 8.3). Statistical analyses were conducted using SAS (version 9.4).

### 2.4 Bioanalytical methods

All plasma and urine PK samples were analyzed for metformin using the validated LC–MS/MS methods at Labcorp Bioanalytical Laboratory (Madison, WI, USA).

In addition, ulotaront and SEP‐383103 were determined by two separate bioanalytical methods for each matrix. For plasma samples collected from the placebo treatment period, only a subset of samples were analyzed for ulotaront to confirm the placebo treatment. The bioanalytical methods used for plasma ulotaront measurements were previously published.^11^, ^12^

### 2.5 Safety and tolerability assessment

The safety assessments were reviewed on a regular basis during the study. Safety assessments include adverse events (AEs), clinical laboratory evaluations (hematology, clinical chemistry, urinalysis), 12‐lead electrocardiograms (ECGs), vital signs measurements, and Columbia‐Suicide Severity Rating Scale (C‐SSRS). AEs were coded using the MedDRA (version 22.0) dictionary by system organ class and PT.

An AE was any untoward medical occurrence that started on or after the first study drug administration up to the final visit. AEs were considered “related” if the relationship to study drug was classified as possible, probable, definite, or was unknown. AEs were considered “not related” if the relationship to study drug was classified as not related.

Vital signs included supine blood pressure, pulse rate, respiratory rate, and body temperature. Vital signs were taken once at screening, once daily while the subject was in‐clinic, once on day of discharge, and once at follow‐up. On dosing days, vital signs were collected pre‐dose of ulotaront or placebo. At screening and admission (Day 2), blood pressure and pulse rate were collected in both supine and standing positions. Orthostatic (supine to standing) vital sign collection was collected if the subject developed symptoms consistent with orthostatic hypotension (e.g., lightheadedness, dizziness upon standing).

Safety ECGs were performed and reviewed prior to the dose of ulotaront or placebo, 3 and 5 h post‐dose of metformin on Day 1 and Day 6. All cardiac safety ECGs were centrally over‐read.

### 2.6 Statistical methods

The primary analysis was conducted using a linear mixed effects model with the natural log‐transformed PK parameter as the dependent variable, treatment (placebo + metformin, ulotaront + metformin), sequence, sex, and period as fixed effects and subject nested within sequence as a random effect. Clinical site was added as a fixed effect. From this model, least squares means for metformin with and without ulotaront and the corresponding two‐sided 90% CIs, the estimated treatment differences and the corresponding 90% CIs were calculated. These log‐transformed results were back transformed to the original scale by exponentiation to obtain the point estimates and the corresponding two‐sided 90% CIs for the geometric means and ratios of the geometric means of the primary PK endpoints between the ulotaront plus metformin and metformin alone.

The primary, secondary, and exploratory PK endpoints (AUC_inf_, AUC_last_, *C*_max_, *t*_1/2_, CL/F, and *V*_Z_/F) for ulotaront and metformin were summarized in Tables 1, 2, 3. Metformin renal PK endpoints (CL_R_, *A*_e_, and *F*_e_) were also summarized in Table 2.

**TABLE 1:** Plasma pharmacokinetic parameters of ulotaront and SEP‐383103.

PK parameters	Unit	Ulotaront				SEP‐383103			
		*N*	Mean (SD)	Geometric mean (CV%)	Median (min, max)	*N*	Mean (SD)	Geometric mean (CV%)	Median (min, max)
*C*_max_	ng/mL	24^a^	367 (123)	348 (35.3)	368 (164, 717)	24^a^	154 (34.9)	150 (22.6)	156 (99.7, 255)
*t*_max_	h	24^a^	NA (NA)	NA	1.51 (0.98, 5.00)	24^a^	NA (NA)	NA	6.00 (3.00, 8.00)
AUC_inf_	ng * h/mL	24^a^	3730 (1030)	3590 (29.3)	3740 (2060, 6240)	23^a^ ^,^ ^b^	2620 (797)	2510 (30.1)	2490 (1550, 4810)
AUC_last_	ng * h/mL	24^a^	3700 (985)	3570 (28.7)	3730 (2050, 5850)	24^a^	2630 (780)	2520 (29.7)	2490 (1540, 4760)
*t*_1/2_	h	24^a^	9.32 (2.42)	9.04 (25.7)	9.39 (5.65, 14.5)	23^a^ ^,^ ^b^	9.17 (1.78)	8.99 (20.7)	9.19 (5.43, 12.2)
CL/F	L/h	24^a^	29.0 (8.78)	27.9 (29.3)	26.8 (16.0, 48.6)	NA	NA	NA	NA
*V*_z_/F	L/h	24^a^	386 (148)	363 (35.4)	348 (190, 807)	NA	NA	NA	NA

Abbreviations: *λ*_z_, terminal elimination rate constant; NA, not available; PK, pharmacokinetic; *R* ^2^_adj_, coefficient of determination for calculation of *λ*_z_.

^a^ One subject vomited within 2 times *t*_max_; PK parameters were calculated but excluded from summaries.

^b^ *R* ^2^_adj_ was <0.700 for one subject. *λ*_z_ and related PK parameters were calculated but excluded from summaries.

**TABLE 2:** Plasma and urine pharmacokinetic parameters of metformin along and in combination with ulotaront.

PK parameters	Unit	Placebo + Metformin‐HCl (850 mg)				Ulotaront (100 mg) + Metformin‐HCl (850 mg)			
		*N*	Mean (SD)	Geometric mean (CV%)	Median (min, max)	*N*	Mean (SD)	Geometric mean (CV%)	Median (min, max)
*C*_max_	ng/mL	24^a^	1230 (416)	1170 (31.8)	1110 (724, 2330)	24^b^	1130 (336)	1080 (31.5)	1030 (542, 1730)
*t*_max_	h	24^a^	NA	NA	4.0 (2.0, 5.0)	24^b^	NA	NA	4.5 (2.0, 12.0)
AUC_inf_	ng * h/mL	23^a^ ^,^ ^c^	10 500 (3370)	10 000 (31.1)	10 100 (5730, 18 200)	22^b^ ^,^ ^d^	11 500 (2830)	11 000 (30.1)	12 500 (4630, 16 100)
AUC_last_	ng * h/mL	24^a^	11 200 (4870)	10 400 (37.7)	10 200 (5710, 28 000)	24^b^	11 000 (2880)	10 500 (31)	11 600 (4560, 16 100)
*t*_1/2_	h	23^a^ ^,^ ^c^	9.80 (5.85)	8.69 (49.7)	8.86 (4.79, 31.5)	22^b^ ^,^ ^d^	15.2 (16.2)	11.6 (75)	10.2 (4.55, 80.4)
CL/F	L/h	23^a^ ^,^ ^c^	68.9 (19.9)	66 (31.1)	65.9 (36.5, 116)	22^b^ ^,^ ^d^	63.0 (23.0)	60 (30.1)	53.2 (41.1, 143)
*V*_z_/F	L	23^a^ ^,^ ^c^	910 (459)	828 (44.5)	844 (411, 2330)	22^e^ ^,^ ^f^	1280 (1230)	1000 (72.5)	1020 (414, 6240)
CL_R_	L/h	24^a^	15.3 (9.07)	10.2 (222)	13.5 (0.147, 35.7)	24^b^	17.2 (10.6)	13.3 (101)	18.3 (1.81, 46.9)
*A*_e last_	mg	23^a^ ^,^ ^c^	162 (84.2)	112 (205)	179 (1.42, 303)	24^b^	183 (108)	141 (106)	200 (19.1, 393)
*F*_e last_	% dose	23^a^ ^,^ ^c^	24.4 (12.7)	16.8 (205)	27 (0.215, 45.7)	24^b^	27.6 (16.3)	21.2 (106)	30.2 (2.89, 59.2)

Abbreviations: *λ*_z_, terminal elimination rate constant; PK, pharmacokinetic; *R* ^2^_adj_, coefficient of determination for calculation of *λ*_z_.

^a^ One subject had a Period 2 predose concentration >5% of *C*_max_; PK parameters were calculated but excluded from summaries and statistical analyses.

^b^ One subject had a Period 2 predose concentration >5% of *C*_max_; PK parameters were calculated but excluded from summaries.

^c^ *R* ^2^_adj_ was <0.700 for one subject; *λ*_z_ and related PK parameters were calculated but excluded from summaries and statistical analyses.

^d^ Two subjects have missing concentration results and/or incomplete urine collections; PK parameters were excluded from summaries.

^e^ One subject vomited within 2 times *t*_max_; PK parameters were calculated but excluded from summaries and statistical analyses.

^f^ *R* ^2^_adj_ was <0.700 for two subjects; *λ*_z_ and related PK parameters were calculated but excluded from summaries and statistical analyses.

## 3 RESULTS

### 3.1 Subjects

A total of 100 subjects were screened for participation, of which 75 (75.0%) subjects were screening failures. The mean age of the study population was 48.1 years (range, 21–61 years). Twenty‐one (84.0%) subjects were male, and 4 (16.0%) subjects were female. Twenty‐four (96.0%) subjects were Black or African American, and 1 (4.0%) subject was White. Two (8.0%) subjects were of Hispanic or Latino ethnicity. The mean body mass index was 28.57 kg/m^2^ at baseline (range, 21.0–35.6 kg/m^2^). All 25 (100.0%) subjects in the study met the Diagnostic and Statistical Manual of Mental Disorders (DSM‐5) criteria for the diagnosis of schizophrenia and were under the subtype of schizophrenia. No subjects had any other psychiatric disorders. Twenty‐one (84.0%) subjects were CYP2D6 normal metabolizers (NM), and 4 (16.0%) subjects were CYP2D6 intermediate metabolizers (IM).

Of the 25 subjects, 20 (80.0%) completed the study. Of the 5 (20.0%) subjects who discontinued the study, 3 (12.0%) subjects discontinued due to AEs, and 2 (8.0%) subjects were lost to follow‐up. One (4.0%) AE discontinuation was related to coronavirus disease (COVID‐19).

### 3.2 Bioanalytical assay performance

Plasma ulotaront and SEP‐383103 were measured by two separate LC–MS/MS methods with the same calibration range from 0.250 to 250 ng/mL.^11^, ^12^ Over this entire study, the precision and accuracy of quality control (QC) samples were found to be 4.0% to 4.9% CV and −3.0% to 4.0% relative error (RE) for ulotaront, and 3.2% to 4.7% CV and 0.0% to 6.0% RE for SEP‐383103, respectively. Moreover, the incurred sample reanalysis (ISR) pass rates of 97.7% for ulotaront and 95.0% for SEP‐383103 demonstrated excellent analytical performance. Plasma metformin was measured by a Labcorp‐owned LC–MS/MS method (2.00–2000 ng/mL). The precision and accuracy of QC samples were 4.3% to 6.7% CV and −4.0% to −0.5% RE, respectively. ISR results showed 98.6% pass rate. Urine metformin was measured by a validated LC–MS/MS method (0.250–250 ng/mL). The precision and accuracy of QC samples were 2.8% to 13.6% CV and 7.0% to −14.7% RE, respectively. ISR results had 83.7% pass rate.

### 3.3 Ulotaront and SEP‐383103 pharmacokinetics

**FIGURE 1:**
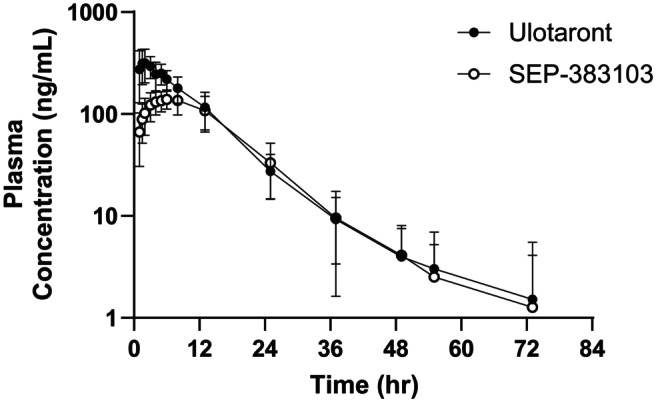
Ulotaront and metabolite SEP‐383103 plasma concentrations after administration of a single dose of 100 mg ulotaront to healthy volunteers. Concentrations are presented as mean ± S.D.

Ulotaront and SEP‐383103 PK following single oral dose administration of 100 mg ulotaront to subjects with schizophrenia are summarized in Table 1, and the plasma concentrations are plotted in Figure 1. Ulotaront was absorbed rapidly in systemic circulation with a median *t*_max_ of 1.5 h. The major but pharmacologically inactive metabolite SEP‐383103 also appeared rapidly but did not reach *C*_max_ until 6 h post‐dose. Exposure to SEP‐383103 represented 71.3% of parent ulotaront exposure based on AUC_inf_. Renal clearance appeared to be a minor elimination pathway for ulotaront with approximately 10% of the administered dose recovered unchanged in urine. Both the ulotaront PK and the SEP‐383103 PK observed in this study are consistent with previously reported results after a single dose of ulotaront.^5^, ^7^, ^11^

### 3.4 Metformin pharmacokinetics

**FIGURE 2:**
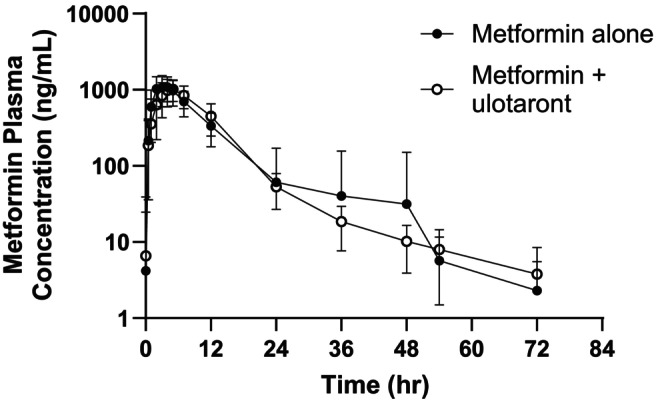
Metformin plasma concentrations after administration of 850 mg metformin alone, and in combination with 100 mg ulotaront. Concentrations are presented as mean ± S.D.

**FIGURE 3:**
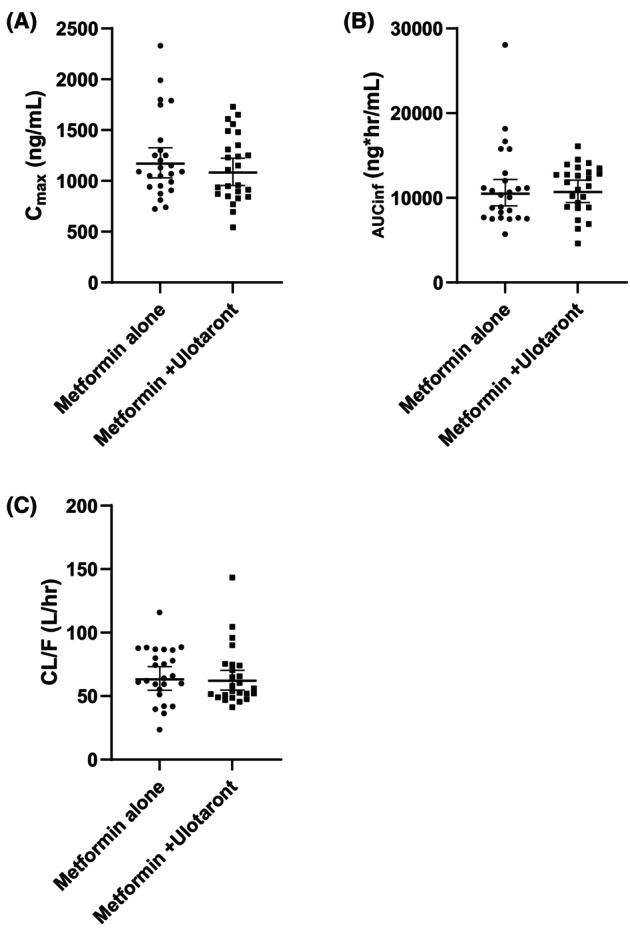
Individual metformin PK parameters after administration of 850 mg metformin alone, and in combination with 100 mg ulotaront. (A) *C*_max_, (B) AUC_inf_, and (C) CL/F were compared between both arms. Lines represented mean ± 90% CI.

Metformin PK parameters after a single dose of 850 mg with and without concomitant 100 mg ulotaront are summarized in Tables 2 and 3, and the plasma concentrations are plotted in Figure 2. Figure 3 shows the *C*_max_, AUC_inf_, and CL/F of each subject. Metformin plasma concentrations were comparable when metformin was administered with and without 100 mg ulotaront. An approximate 10% reduction in CL/F was noted in the combination treatment; however, the resulting changes in metformin plasma exposure parameters (10.02% decrease in *C*_max_ and 10.63% increase in AUC_inf_) were not statistically significant. The geometric mean ratios (metformin with and without concomitant 100 mg ulotaront) were 89.98% and 110.63% for *C*_max_ and AUC_inf_, respectively, with the 90% CIs completely within 80%–125% for both primary endpoints (Table 3).

**TABLE 3:** Effect of ulotaront on metformin exposure.

PK parameter	Unit	*N*	Geo LS mean		Geo LS mean ratio (%)	90% CI of Geo LS mean ratio (%)	Treatment effect *p* value
			Placebo + metformin	Ulotaront + metformin			
*C*_max_	ng/mL	23^a^	1230	1110	89.98	81.47, 99.38	.082
AUC_inf_	ng * h/mL	20^a^ ^,^ ^b^	11 300	12 500	110.63	100.99, 121.18	.071

Abbreviation: Geo LS, geometric least squares.

^a^ One subject had a Period 2 predose concentration >5% of *C*_max_, and one subject vomited within 2 times *t*_max_; PK parameters were calculated but excluded from summaries and statistical analyses.

^b^ *R* ^2^_adj_ was <0.700 for 3 subjects, *λ*_z_ and related PK parameters were calculated but excluded from summaries and statistical analyses.

Metformin renal clearance was also determined. Ulotaront treatment increased the geometric mean CL_R_ from 10.2 to 13.3 L/h. However large intersubject variation was observed (geometric mean CV >100%) for renal clearance parameters CL_R_, *A*_e_, and *F*_e_ (Table 2). Because of the large variation, a clear trend of renal clearance due to ulotaront treatment was not discernable. While ulotaront treatment decreased metformin renal clearance in some subjects, it increased metformin renal clearance in other subjects.

### 3.5 Safety and tolerability

Ulotaront was generally well tolerated when administered as a single dose of 100 mg with a single dose of metformin‐HCl 850 mg. There were no AEs leading to death during the study. One subject experienced a severe AE (SAE) of asthma (reported term: exacerbation of asthma) that led to discontinuation from the study. The Investigator assessed asthma as severe and not related to the study drug. There were 2 additional discontinuations, with one subject discontinued after diagnosis with a coronavirus infection (reported term: COVID‐19 infection) and the other subject discontinued due to anxiety (reported term: increased anxiety). The Investigator assessed the coronavirus infection as mild and the anxiety as moderate, and both were assessed as not related to the study drug. The most common (i.e., more than one subject) AEs experienced in subjects receiving ulotaront + metformin were anxiety, nausea, sedation, and vomiting, which are consistent with the known safety profile of ulotaront in subjects with schizophrenia. One subject experienced an AE of hypoglycemia on Day 1 after receiving ulotaront + metformin, which resolved on the same day. The Investigator assessed the hypoglycemia as moderate and not related to the study drug; the subject had declined lunch on Day 1 and the etiology of the hypoglycemia was low caloric intake and metformin administration. There were no clinically meaningful changes in vital signs or ECG findings. There were no positive findings on the C‐SSRS for the study.

## 4 DISCUSSION

OCT2 is a renal transporter and plays a role in the renal elimination of organic anion and cation drugs.^13^, ^14^ OCT2‐mediated DDI have been reported^9^, ^15^, ^16^, ^17^, ^18^, ^19^; however, most of the reported clinical DDIs are likely mediated by both OCT2 and MATEs, since the victims and/or the perpetrators from these studies are substrates or inhibitors of both OCT2 and MATEs.

Relatively specific inhibitors for MATE1 and MATE2K such as famotidine, nizatidine and pyrimethamine, and relatively specific inhibitors for OCT2 such as disopyramide, have been identified.^20^, ^21^, ^22^, ^23^ Unlike most OCT2 and MATEs inhibitors, ulotaront is an inhibitor of OCT2 but not MATE1 or MATE2K.^6^ Ulotaront unbound *C*_max_ at steady state after repeat dose of 100 mg is in the range of 1.2–1.5 μM, and the unbound *C*_max_/IC_50_ ratio is greater than 0.1, which suggests the DDI is likely according to FDA guidance on in vitro drug interaction studies.^8^ Although OCT2‐mediated DDI potential with ulotaront as the perpetrator was assessed as remote via PBPK simulation, given the knowledge gap in predicting transporter DDI via PBPK, a clinical study was conducted.

The current study was designed to evaluate whether a single dose of 100 mg ulotaront would affect the PK of metformin, an index substrate commonly used as a substrate for OCT2‐ and MATE‐mediated DDI studies.^9^, ^19^, ^21^, ^23^, ^24^, ^25^ A single dose of 100 mg ulotaront was selected because 100 mg is the anticipated maximum therapeutic dose, little or no accumulation of ulotaront upon multiple dose administration was observed in human subjects,^5^ and OCT2 inhibition is not anticipated to be time dependent.

In general, OCT2/MATEs‐mediated DDI with metformin as the victim is moderate with less than 2‐ to 3‐fold increase in metformin exposures.^16^, ^19^, ^23^, ^24^, ^25^ Although metformin renal elimination is mediated by both OCT2 and MATEs, DDIs that are predominately determined by MATEs inhibition or by OCT2 inhibition have been observed. While the DDI between cimetidine and metformin is mainly due to MATEs inhibition,^26^, ^27^ the DDI between dolutegravir and metformin is largely caused by OCT2 inhibition because dolutegravir is a much more potent inhibitor of OCT2 over MATEs.^28^ Co‐administration of 50 mg dolutegravir once a day (qd) or twice a day (bid) led to 79% and 145% increase of metformin AUC, respectively.^9^ Compared to the unbound *C*_max_/IC_50_ ratio of dolutegravir (approximately 2–3.6) after oral administration at 50 mg qd or bid,^9^, ^28^ the unbound *C*_max_/IC_50_ ratio of ulotaront after oral administration at 100 mg qd is only about 0.8; therefore, less DDI is anticipated. The impact of ulotaront co‐administration on metformin PK was simulated via physiologically based pharmacokinetic (PBPK) approach (to be published). The simulations project that co‐administration of ulotaront results in approximately 1.1‐fold increase in metformin exposure based on in vitro studies indicating that ulotaront has the potential to inhibit OCT2 only; however, an approximately 1.4‐fold increase of metformin exposure was projected when the simulations were conducted using a 10‐fold lower value for OCT2 IC_50_ as compared to the experimentally determined value, or assuming ulotaront inhibits both OCT2 and MATEs. The simulations suggest that ulotaront could have affected metformin PK if it was a more potent inhibitor of both OCT2 and MATEs.

Ulotaront was generally well tolerated when administered as a single dose of 100 mg with a single dose of 850 mg metformin‐HCl. The observed ulotaront and SEP‐383103 PK from the current study (Table 1; Figure 1) are consistent with the PK observed in previous clinical trials for ulotaront.^5^

The observed metformin PK from this study is also consistent with reported metformin PK at the same dose to healthy volunteers.^29^, ^30^, ^31^ As summarized in Table 3, co‐administration of 100 mg ulotaront has no statistically significant effect on the PK based on *C*_max_ and AUC_inf_. The geometric least squares mean ratios were 89.98% and 110.63%, respectively, with 90% CIs contained within 80%–125% for both endpoints. Although the *t*_max_ is slightly increased, the median *t*_max_ was comparable with and without co‐administration of 100 mg ulotaront. The reason for the slight shift in *t*_max_ is not known yet.

While most the of subjects enrolled in this study are Black or American African and are predominantly male, a population PK analysis indicated that race or sex did not have a meaningful impact on ulotaront PK.^5^ Likewise, there were no apparent clinically relevant impacts of race or sex on metformin PK.^32^, ^33^ Therefore, the enrollment of subjects in this study will unlikely cause any bias in the data interpretation.

## 5 CONCLUSIONS

A single dose of 100 mg ulotaront was considered to be safe and generally well tolerated when co‐administered with a single dose of 850 metformin‐HCl to male and female subjects with schizophrenia.

The highest anticipated clinical dose of ulotaront (100 mg) has no statistically significant effect on the PK of a single dose of metformin based on *C*_max_ and AUC_inf_. Co‐administration of ulotaront is not expected to require dose adjustment of metformin or other drugs cleared by OCT2.

## AUTHOR CONTRIBUTIONS

All authors contributed equally and all were involved in the study design, data acquisition, or data analysis/interpretation and in drafting or critically revising the manuscript. All authors reviewed the final version of the manuscript and gave approval for submission.

## FUNDING INFORMATION

Funding was provided by Sumitomo Pharma America, Inc. and Otsuka Pharmaceutical Development & Commercialization, Inc.

## CONFLICT OF INTEREST STATEMENT

Guangqing Xiao, Yu‐Luan Chen, Lei Shi, Seth C. Hopkins, and Gerald R. Galluppi are employees of Sumitomo Pharma America, Inc. (“SMPA”). Hironobu Tsukada is a former employee of SMPA. SMPA discovered ulotaront in collaboration with PsychoGenics based in part on a mechanism‐independent approach using the in vivo phenotypic SmartCube® platform and associated artificial intelligence algorithms.

## ETHICS STATEMENT

The study protocols were approved by institutional review boards at the respective sites, and patients/guardians provided informed written consent in accordance with the current revision of the Declaration of Helsinki.

## PATIENT CONSENT STATEMENT

Patients provided written informed consent.

## Data Availability

Access to de‐identified participant data will be provided after a research proposal is submitted online (https://vivli.org) and receives approval from the Independent Review Panel and after a data sharing agreement is in place.
